# Molecular Identification, Histopathology and Antibiotic Susceptibility Profiling of *Aeromonas veronii* Isolated from *Oreochromis niloticus* in Bangladesh

**DOI:** 10.1002/vms3.70103

**Published:** 2024-11-05

**Authors:** Sayed Mashequl Bari, Mohammad Muttakinul Islam, Aktia Amina, Marufa Khatun, A. M. Shahabuddin

**Affiliations:** ^1^ Department of Aquatic Animal Health Management Sher‐e‐Bangla Agricultural University Dhaka Bangladesh; ^2^ Fish Disease Laboratory Department of Aquatic Animal Health Management Sher‐e‐Bangla Agricultural University Dhaka Bangladesh; ^3^ Department of Aquaculture Sher‐e‐Bangla Agricultural University Dhaka Bangladesh

**Keywords:** *Aeromonas veronii*, antibiotic susceptibility test, histopathology, molecular identification, Tilapia

## Abstract

**Background:**

Tilapia (*Oreochromis niloticus*) is the most widely cultured freshwater fish species in Bangladesh and worldwide. However, commercial tilapia culture systems face increasing challenges from bacterial infections.

**Objectives:**

The objective of this study was to identify the bacterial isolates from infected tilapia in an intensive cage culture farm located along the Shitalakshya River in Bangladesh.

**Methods:**

Infected fish samples were collected and underwent comprehensive clinical and post‐mortem investigations, followed by phenotypic, biochemical and molecular identification of the bacterial isolates, as well as histopathological and antibiotic susceptibility examinations.

**Results:**

Phenotypic and biochemical characterization showed similarities of the ‐collected isolates with *Aeromonas veronii*. Moreover, molecular analysis of the bacterial conserved region 16S rRNA also confirmed these isolates as *A. veronii*. The analysed 16S rRNA sequence (GenBank accession no. PP832815) showed a close relationship (100% identity) with *A. veronii* from China (GenBank accession no. MT071624) in the NCBI BLAST search, and in the phylogenetic tree, they grouped in a single clade. This close genetic relationship is also supported by the low genetic distance between the isolates. Histopathological analysis revealed gross pathological changes like necrosis, hypertrophy and inflammation in muscle tissues. The isolates were found to be sensitive to multiple antibiotics but resistant to trimethoprim and sulphamethoxazole.

**Conclusion:**

This study investigated the presence of *A. veronii* infection in tilapia (*O. niloticus*) in an intensive cage culture farm in Bangladesh.

## Graphical abstract

*Aeromonas veronii* is a pathogenic bacterium that causes infections in tilapia (*Oreochromis niloticus*). This study identifies *A. veronii* as the cause of mortality in Nile tilapia in intensive cage culture on the Shitalakshya River, Bangladesh. Infected fish underwent clinical, histopathological, biochemical, and molecular analyses. Molecular identification via 16S rRNA confirmed the isolates as *A. veronii*. Histopathology revealed severe tissue damage, and antibiotic susceptibility tests indicated resistance to trimethoprim and sulfamethoxazole.

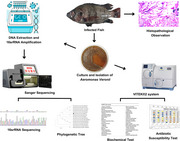

## 1 Introduction

Tilapia (*Oreochromis niloticus*) is an intensively cultured freshwater fish species in Bangladesh and worldwide (Baumgartner, Ford and Hanson, 2017; Abd El‐Naby et al. 2024). Tilapia cage culture is a widely practiced method for high‐volume fish production in tropical regions (Gorlach‐Lira et al. 2013; da Rocha Brande et al. 2023). In Bangladesh, different fish species were cultured in floating cages, including Pabda (*Ompok bimaculatus*) (Kunda et al. 2022), Pangas (*Pangasius pangasius*) (Ara et al. 2020), *O. niloticus* (Begum et al. 2017; Hossain et al. 2017; dos Santos et al. 2023), Cyprinid (Ran et al. 2018) and climbing perch (*Anabas testudineus*) (Habib et al. 2015). Fluctuations in water quality in open water bodies is result from the external factors like polluted runoff and industrial waste (Alam et al. 2022). These fluctuations can have negative effects on the growth and production of organisms, making them more vulnerable to pathogenic microorganisms (da Rocha Brande et al. 2023).

Tilapia have been found to be infected with various pathogenic bacteria in intensive aquaculture systems, including species of the genera *Vibrio*, *Aeromonas* (Azzam‐Sayuti et al. 2021), *Pseudomonas* and *Streptococcus* (Bekele et al. 2019). *Aeromonas* spp. belongs to the family Aeromonadaceae (Pessoa et al. 2019) are Gram‐negative, facultative anaerobic bacteria that can be found in freshwater, estuarine and marine environments (Janda et al. 2010). Different *Aeromonas* species, including *Aeromonas hydrophila* (Pauzi et al. 2020), *Aeromonas sobria*, *Aeromonas caviae*, *Aeromonas jandaei* (Dong et al. 2017), *Aeromonas dhakensis* (Carriero et al. 2016) and *Aeromonas veronii* (Dong et al. 2017), are responsible for septicaemic diseases in different fish species.

Among the pathogenic *Aeromonas* bacteria, *A. veronii* appears to have the widest range of hosts in terms of virulence (Lü et al. 2016; Rahman et al. 2002). In tropical freshwater areas, the disease Motile *Aeromonas* Septicaemia (MAS) caused by *Aeromonas* spp. affects various cultured (Li et al. 2019; Monir et al. 2020) and open water fish species (Saharia et al. 2020). This disease leads to high mortality rates and is characterised by clinical signs such as skin and fin ulcers, the accumulation of body fluid in the abdomen (ascites) and eye (exophthalmia) (Dong et al. 2017; Janda et al. 2010). Fish infected with *Aeromonas* spp. can suffer from acute and chronic diseases, such as haemorrhagic septicaemia and skin ulcers (Beaz‐Hidalgo and Figueras 2013).

Dong et al. (2015) demonstrated that both alpha and beta‐haemolytic *A. veronii* strains exhibited significant levels of pathogenicity towards tilapia fingerlings. Tilapia infected by *A. veronii* showed lethargy, ulcerations, pale spots and haemorrhages along their body (Dong et al. 2017; Hassan et al. 2017). It is observed that the excessive and inefficient use of different antibiotics to treat *Aeromonas* infections in aquaculture has led to antimicrobial resistance (AMR) against multiple groups (Aravena‐Román et al. 2012). Many studies have identified bacterial infections in tilapia across various aquaculture systems. However, research on bacterial infections, particularly in cage aquaculture system, remains limited (Chitmanat et al. 2016). This study aimed to identify bacterial isolates from infected *O. niloticus* in a cage culture farm along the Shitalakshya River in Bangladesh. To accomplish this objective, we employed phenotypic, biochemical, histopathological and molecular techniques. Furthermore, we assessed the antibiotic sensitivity of identified bacterial isolates.

## 2 Materials and Method

### 2.1 Collection of Infected Fish Samples

**FIGURE 1:**
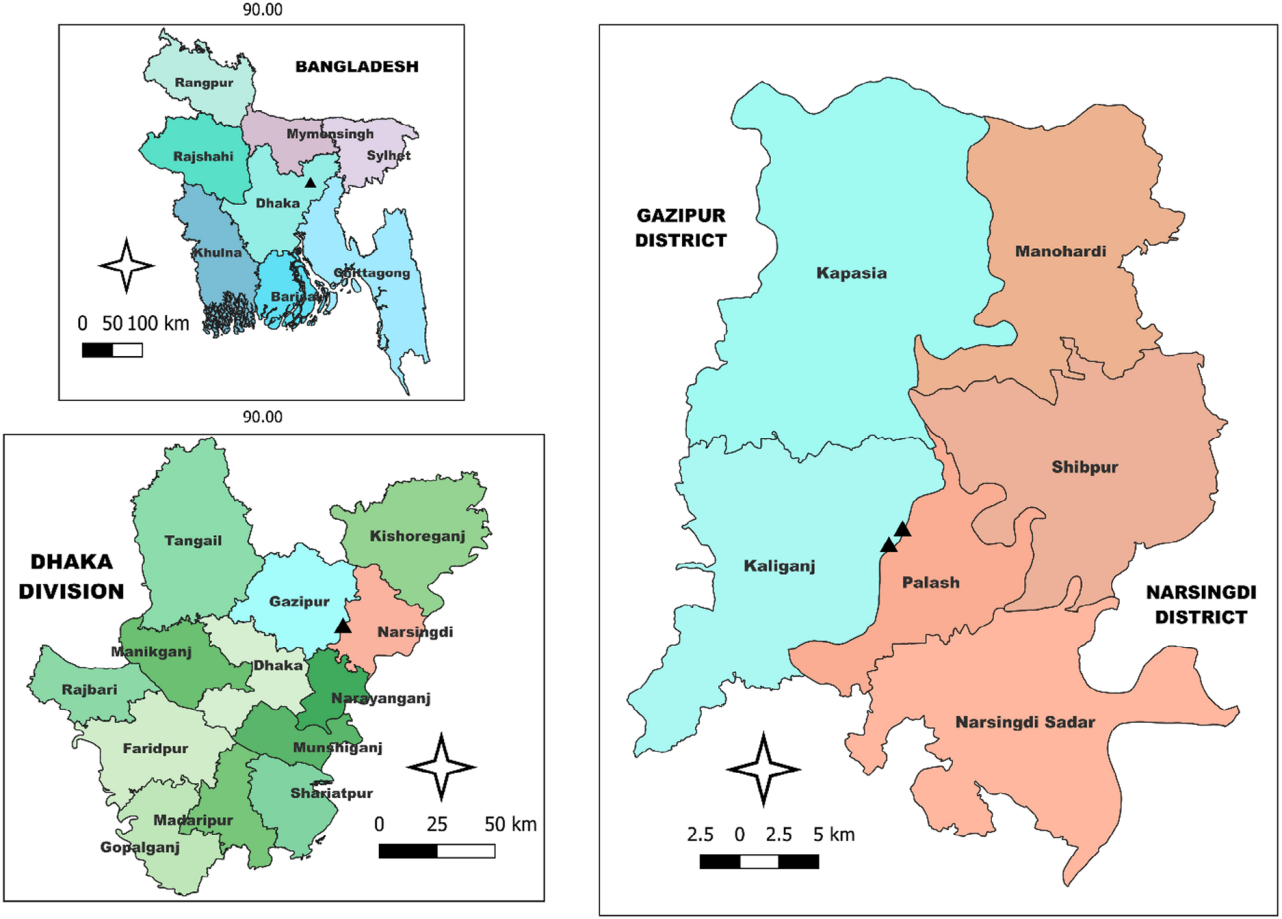
Geographical map of the sampling area (software: QGIS 3.38). Black points on the map represent the sampling points on the country, division and district maps.

In June 2023, a total of 40 infected and moribund Tilapia samples were collected from a floating cage fish farm located in the Shitalakshya River, Polash upazila, Narsingdi district, Bangladesh. The two sampling areas were (23.97780° N, 90.63149° E and 23.97734° N, 90.63122° E) depicted in Figure 1 using QGIS 3.38. The collected fish samples showed a range of clinical signs, including exophthalmia, abdominal swelling, haemorrhages on the skin and around the anus, along with swollen and fragile kidneys and spleens. The infected samples were transported to the fish disease laboratory at Sher‐e‐Bangla Agricultural University, Dhaka, Bangladesh through proper icing and transportation. Infected samples were sacrificed following the ethical guidelines of the Sher‐e‐Bangla Agricultural University research ethics committee for the use of animals.

### 2.2 Isolation, Culture and Phenotypic Identification of the Pathogen

Prior to collecting bacteriological samples, the outer body surfaces of the fish samples were examined and sterilised using 70% ethanol. After sterilisation, the post‐mortem examination protocol was outlined by Noga (2010). The fishes were dissected in a sterile manner, and various organs and body parts, such as the eye, muscle, gill and liver were collected. The tissue samples were then pooled and homogenised using a mortar and pestle. Next, the samples were inoculated onto a selective medium MacConkey agar to isolate Gram‐negative and non‐lactose fermenting bacteria. The plates were then incubated at 37°C for 24–48 h, under aerobic conditions. To obtain a pure culture, dominant colonies were sub‐cultured on the blood agar media at 37°C for 24–48 h. Then the VITEK 2 compact system (BioMérieux Inc., Hazelwood, MO) (Moehario et al. 2021) GN (Gram‐negative fermenting and non‐fermenting bacilli) reagent card was used for the identification of isolates. The final identification was performed using the VITEK 2 automated compact system with GN card, which contained 47 biochemical tests against Gram‐negative bacteria.

### 2.3 Molecular Identification of the Pathogen

To extract DNA, four colonies were inoculated in nutrient broth and incubated overnight at 37°C. Each sample (1.5 mL) was then centrifuged (13000 rpm for 10 min), and the cells were lysed with lysis buffer (350 µL) and proteinase K (200 mg/mL), followed by incubation at 65°C for 30 minutes. After cooling, RNase A (3 µL) was added and incubated at 37°C for 10 min. Then equal volume (373 µL) of phenol: chloroform alcohol (25:24:1) was mixed in, centrifuged (1400 rpm for 10 min) and the aqueous layer (about 200 µL) was transferred to a fresh microcentrifuge tube. Then equal volume of isoamyl alcohol (24:1) added to the tube, centrifuged (1400 rpm for 10 min) and transfered the upper layer (150 µL) to a new eppendrof tube. The DNA was then precipitated with ice‐cooled ethanol (800 µL), stored at −40°C for 1h and centrifuged (13000 rpm for 10 min). Then the DNA pellet was washed with chilled 70% ethanol (1000 µL) containing ammonium acetate (20 µL of 7.5 M) and keep at ‐20°C for 30 min. Then centrifuged (13000 rpm for 10 min), air‐dried and dissolved in TE buffer (50µL, pH 7.6) by heating at 37°C for 10 min.

After DNA extraction, PCR (polymerase chain reaction) amplification of 16S rDNA gene (Acinas et al. 2004) sequences were performed using 16S rRNA universal primer sets: forward 27F (5′‐AGAGTTTGATCCTGGCTCAG‐3′) and reverse 1492R (5′‐TACGGYTACCTTGTTACGACTT‐3′). For each sample tube, the reaction volume of the PCR was 25 µL, which included 10 ng of template DNA, 2 mM MgCl_2_, 1 mL of each primer (10 mM), and 1X Taq Master Mix. The size of the DNA amplicon was about 1400 bp. Then the reaction mixtures containing PCR tubes were preheated at 95°C for 5 min in the thermocycler (Applied Biosystems GeneAmp 9700 PCR System) to ensure the denaturation of all DNA templates. The PCR reaction then continued with the following temperature and timespan: one cycle of preheating at 95°C for 5 min, followed by 32 cycles of denaturation at 94°C for 30 s, annealing at 54°C for 30 s, and extension at 72°C for 45 s. This was followed by one cycle of final extension at 72°C for 10 min, and finally, a hold at 4°C overnight. A 1 Kb Plus DNA ladder (New England Biolabs, UK) was used to compare the bands on an agarose gel using a gel documentation system (Bio‐Rad). The PCR products were confirmed on agarose gels (1%) stained with ethidium bromide and visualised using the gel documentation system (EZEE Clearview UV transilluminator). Then purified using a PCR Purification Kit following the manufacturer's instructions.

The four purified products were sequenced using the forward primer (5′‐AGAGTTTGATCCTGGCTCAG‐3′) and reverse primer (5′‐TACGGYTACCTTGTTACGACTT‐3′) in Sanger sequencing (Sanger, Nicklen, and Coulson 1977) at Wuhan Tianyi Huayu Gene Technology Co. Ltd., China. The obtained partial DNA sequences of the 16S rRNA region were edited using BioEdit software, and a well‐trimmed sequence was submitted to the National Center for Biotechnology Information (NCBI) (https://blast.ncbi.nlm.nih.gov/Blast.cgi) GenBank database. The sequence was compared with the available sequences in the NCBI GenBank database using the NCBI Basic Local Alignment Search Tool (BLAST) (McGinnis and Madden 2004). A neighbour‐joining tree (Saitou and Nei 1987) was generated using MEGA 11 software (Tamura, Stecher, and Kumar 2021) based on the Tamura–Nei substitution model (Tamura and Nei 1993) with 1000 bootstrap values (Felsenstein 1985).

### 2.4 Histopathological Study of Infected Tissues

Muscle tissue samples were collected from the skin lesion areas of four severely affected Nile tilapia. The tissue samples were  carefully excised, washed and immediately preserved in buffered 10% formalin for 24 h to ensure proper fixation. The samples were then dehydrated through a graded series of ethanol (50%, 70%, 90% and 100%), followed by 2‐propanol, cleared in xylene and embedded in paraffin wax. Thin serial sections (5 µm) were cut using a rotary microtome (MicroTec, Germany), placed in a hot water bath and transferred to an oven for drying. The sections were then re‐cleared in xylene, stained with haematoxylin and eosin and mounted using DPX. Finally, the stained sections were examined and photographed under a compound microscope (Euromex D. 1355 F050, Netherlands) to observe histopathological changes.

### 2.5 | Antibiotic Susceptibility Test

Antibiotic sensitivity analysis was conducted using the VITEK 2 system (BioMérieux Inc., Hazelwood, MO) with antibiotic susceptibility testing (AST) cards. The antimicrobial susceptibility patterns of the isolated pathogens were determined using a panel of antimicrobials, including piperacillin/tazobactam, cefuroxime, ceftriaxone, cefoperazone/sulbactam, cefepime, imipenem, meropenem, amikacin, gentamicin, ciprofloxacin and trimethoprim/sulphamethoxazole in the VITEK 2 machine (Sun et al. 2021).

## 3 Results

### 3.1 Clinical Signs of Diseased Fish

**FIGURE 2:**
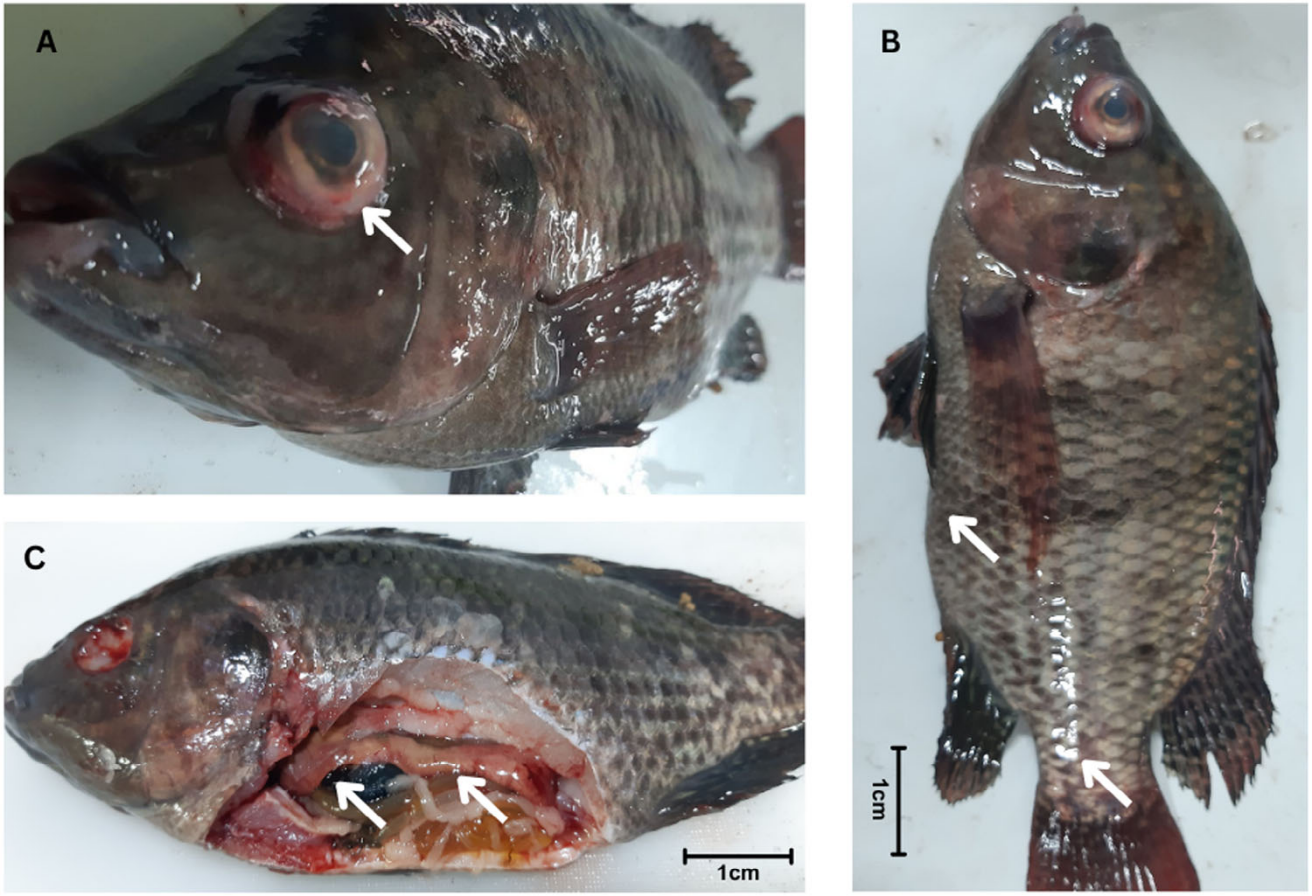
Clinical observation of an infected Tilapia sample. The white arrow indicates (A) exophthalmia in the eye, (B) abdominal distension and black spot on body surface, (C) necrotic spleen, liver and intestine.

The infected fish exhibited abnormal behaviours, such as lethargy and erratic swimming. The external post‐mortem examination revealed exophthalmia with corneal opacity, haemorrhage in the eyes (Figure 2A), darkened skin, abdominal distention and scale loss (Figure 2B). Internally, the post‐mortem examination showed reddish‐yellow ascitic fluid in the abdomen and haemorrhagic visceral organs (Figure 2C).

### 3.2 Phenotypic and Biochemical Identification of the Isolates

**FIGURE 3:**
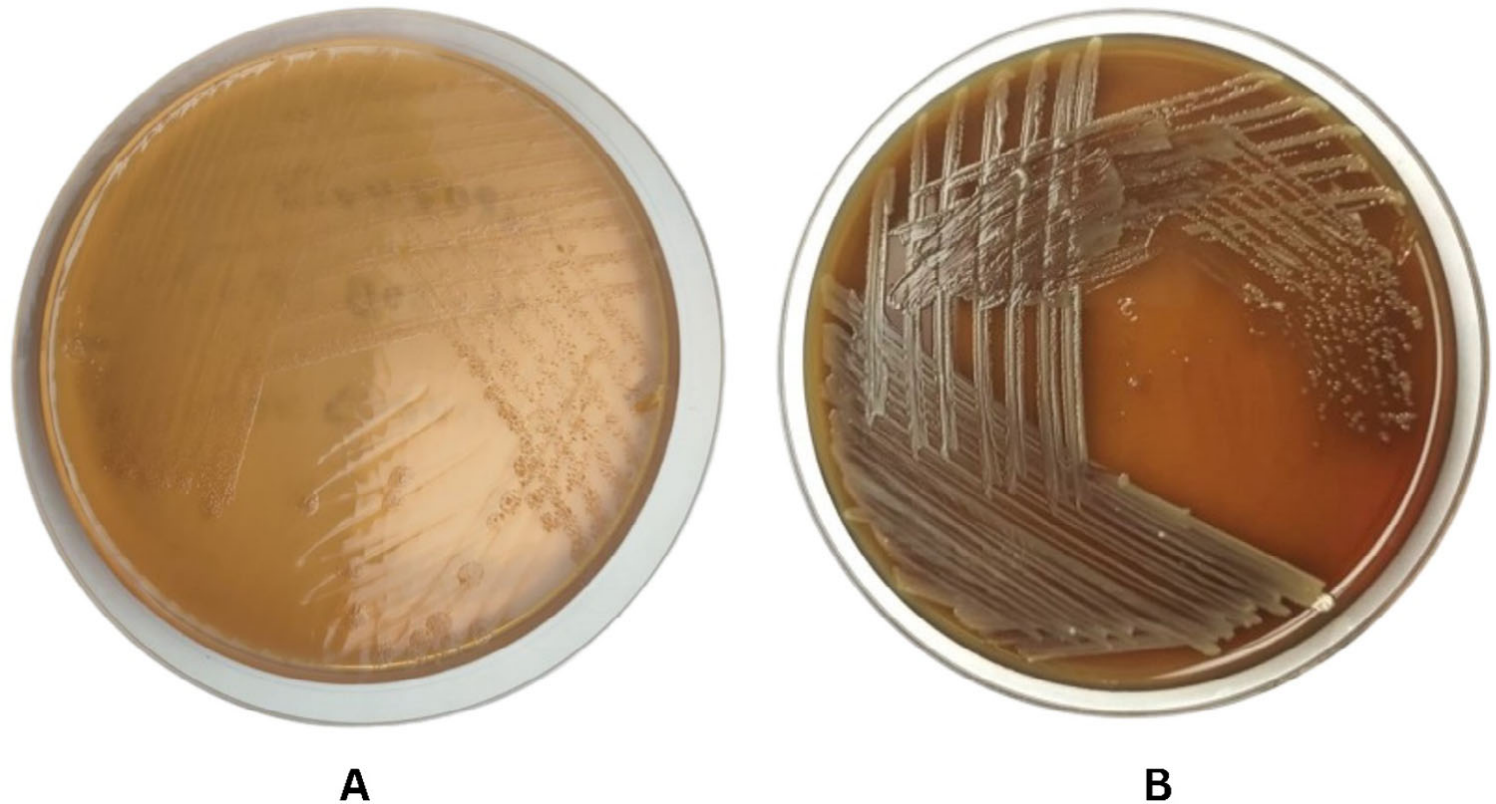
Colony morphology of *Aeromonas veronii*. (A) *A. veronii* produces white colonies on MacConkey agar. (B) Pale smooth, convex and rounded colonies on blood agar.

The initial identification involved assessing physiological traits, motility and growth characteristics. Motility was observed under a microscope. In selective culture, the colonies appeared pale‐shaped on the MacConkey agar (Figure 3A). In sub‐culture, creamy‐white circular, smooth, pure convex colonies up to 3 mm in length were observed on blood agar (Figure 3B). The phenotypic characteristics of the four isolates were confirmed as *A. veronii* (97% probability) using the VITEK 2 compact system. The results of the VITEK 2 biochemical tests also served as complementary characteristics to the initial diagnosis.

The biochemical test results from VITEK 2 show a combination of positive and negative reactions (Table 1). The bacteria tested positive for enzymes and sugar fermentation, including β‐galactosidase, d‐cellobiose and others. However, it tested negative for several decarboxylase activities and certain sugar fermentations, indicating a specific metabolic and enzymatic profile characteristic of *A. veronii*. These phenotypic and biochemical analyses collectively confirmed the isolates as *A. veronii*.

**TABLE 1:** Biochemical characteristics of isolated bacteria from infected tilapia (*Oreochromis niloticus*).

Well biochemical test	Result	Well Biochemical Test	Result
Ala‐Phe‐Pro arylamidase (APPA)	+	Saccharose/sucralose (SAC)	+
Adenosyl (ADO)	−	d‐Tagatose (TAG)	−
l‐Pyrrolidonyl‐arylamidase (PyrA)	−	d‐Trehalose (dTRE)	+
I‐arabitol (IARL)	−	Citrate (sodium) (CIT)	−
d‐Cellobiose (dCEL)	+	Malonate (MNT)	−
β‐Galactosidase (BGAL)	+	5‐keto‐d‐gluconate (5KG)	−
Hydrogen sulphide/H_2_S production (H_2_S)	−	l‐Lactate alkalinization (ILATK)	+
β‐N‐Acetyl‐glucosaminidase (BNAG)	+	α‐Glucosidase (AGLU)	+
Glutamylarylamidase (AGLTP)	−	Succinate alkalinization (SUCT)	+
d‐Glucose (dGLU)	+	β‐N‐acetylgalactosaminidase (NAGA)	−
γ‐glutamyl‐transferase (GGT)	+	α‐Galactosidase (AGAL)	−
Fermentation glucose	+	Phosphatase (PHOS)	−
β‐Glucosidase (BGLU)	+	Glycine arylamidase (GlyA)	+
d‐Maltose (dMAL)	+	Ornithine decarboxylase (ODC)	−
d‐Mannitol	+	Lysine decarboxylase L (LDC)	−
d‐Mannose (dMNE)	−	Histidine assimilation (IHISA)	−
β‐Xylosidase (BXYL)	−	Coumarate (CMT)	+
β‐Alaninearylamidase pna (BAlap)	−	β‐Glucuronidase (BGUR)	−
l‐Proline arylamidase (ProA)	+	O/129 resistance (0129R)	+
Lipase (LIP)	+	Glu‐Gly‐Arg‐arylamidase L (GGAA)	+
Palatinose (PLE)	−	MALATE assimilation (IMLTa)	+
Tyrosine (TyrA)	+	Ellman (ELLM)	+
Arylamidase urease (URE)	−	l‐Lactate assimilation (ILATA)	−
d‐Sorbitol (dSOR)	−		

### 3.3 Molecular Identification Using 16S rRNA

The partial 16S rRNA gene sequence was initially analysed using NCBI BLAST for molecular identification. This was done to assess its similarity to known sequences in the NCBI database. The NCBI BLAST result of the conserved 16S rRNA gene revealed that the studied bacterial strain exhibited a significant level of genetic similarity (100% identity) with *A. veronii*. This species has been found in different fish hosts and geographic locations, such as Bangladesh, India, China, Lithuania, South Korea, Thailand and Uruguay (Table 2).

**TABLE 2:** NCBI BLAST results, sequenced with the 16S rRNA region, are represented in the phylogenetic analysis alongside their host species, GenBank accession numbers, percent identity and geographical locations.

SN	Strain	Host	Accession	Base pair	Indent (%)	Location
1.	*Aeromonas veronii* strain (present study)	*Oreochromis nilotius*	PP832815	1386	100	Bangladesh
2.	*A. veronii* strain LCR7	*Labeo rohita*	MT226399	1413	100	India
3.	*A. veronii* strain NBH	*Carassius auratus*	MT071624	1440	100	China
4.	*A. veronii* strain KR2‐9	Gypsium karst lakes	MN752428	1505	100	Lithuania
5.	*A. veronii* strain PCG1	*Procambarus clarkii*	MN581681	1439	100	China
6.	*A. veronii* strain ATCC 35624	*Silurus asotus*	MK990549	1485	100	South Korea
7.	*A. veronii* strain BN 2054	*Betta splendens*	MG438512	1508	100	Thailand
8.	*A. veronii* strain FZ2N	*Paramisgurnus dabryanus*	MF716720	1433	100	China
9.	*A. veronii* strain XG3‐1‐1	Grass carp pond	MF716697	1437	100	China
10.	*A. veronii* strain v4	*Acipenser baerii*	MF629678	1439	100	Uruguay
11.	*A. veronii* strain BLB‐01	Fish	MF370515	1480	100	India
12.	*A. veronii* strain PYLGC	*Ctenopharyngodon idella*	KY767542	1424	100	China
13.	*A. veronii* strain AnguAV2	*Eel*	PP095387	1479	100	China
14.	*Bacillus subtilis* strain MRS3	Fish	KP659617	1125	—	Spain
15.	*B. subtilis* strain MRS11	Fish	KP659618	1153	—	Spain

**FIGURE 4:**
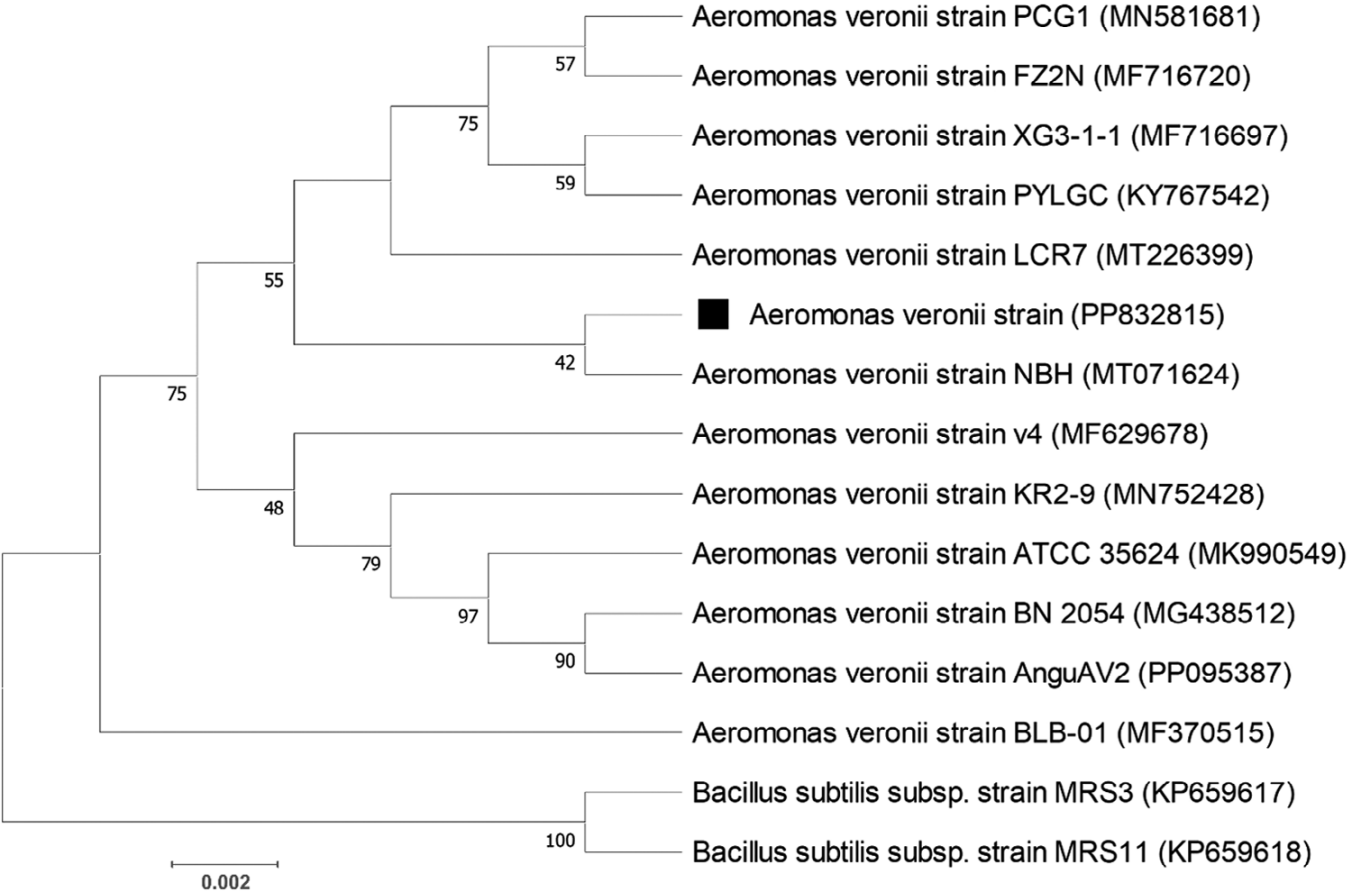
Phylogenetic tree derived from 16S rRNA gene sequences showing relationships of strain PP831815 to all other described closely related species of the same genus *Aeromonas* and out‐group *Bacillus*. The tree was reconstructed using the neighbour‐joining method. Percentage bootstrap values (1000 replicates) are shown at each branch point. The scale bar 0.002 represents substitutions per nucleotide position. The black squared dot represents the studied isolate.

The phylogenetic tree analysis indicates that the studied sample *A. veronii* (GenBank accession no. PP832815) forms a distinct clade with *A. veronii* strain NBH (GenBank accession no. MT071624) of China. This relationship is strongly supported by a 100% bootstrap value (Figure 4). Furthermore, the sample is closely related to strains with GenBank accession numbers MN581681, MF716720, MF716697, KY767542 and MT226399, all of which belong to the Aeromonadaceae family. To provide reference points for rooting the tree and understanding the evolutionary connections between the families under study, the phylogenetic tree also includes the Bacillaceae family (GenBank accession no. KP659617 and KP659618) as out‐groups.

The genetic distance matrix, based on 13 closely related sequences and 2 out‐group sequences, shows that the strains of studied *A. veronii* have very low genetic distances among each other (Table 3). In fact, most pairs have a distance of 0.00 or 0.01, indicating that they are nearly genetically identical. In addition, strains from different geographical locations and hosts show minimal genetic variation. On the other hand, the *Bacillus subtilis* strains MRS3 and MRS11 have significantly higher genetic distances (0.28–0.29) compared to the *A. veronii* strains. This clearly highlights a genetic distinction between these two species.

**TABLE 3:** The genetic distance matrix estimates the evolutionary divergence among the studied isolate and similar *Aeromonas veronii* sequences.

	1	2	3	4	5	6	7	8	9	10	11	12	13	14	15
*A. veronii* (PP832815)															
*A. veronii* strain LCR7 (MT226399)	0.00														
*A. veronii* strain NBH (MT071624)	0.00	0.00													
*A. veronii* strain KR2‐9 (MN752428)	0.00	0.00	0.01												
*A. veronii* strain PCG1 (MN581681)	0.00	0.00	0.01	0.01											
*A. veronii* strain ATCC 35624 (MK990549)	0.00	0.00	0.01	0.00	0.01										
*A. veronii* strain BN 2054 (MG438512)	0.00	0.00	0.01	0.00	0.01	0.00									
*A. veronii* strain FZ2N (MF716720)	0.00	0.00	0.01	0.00	0.00	0.00	0.00								
*A. veronii* strain XG3‐1‐1 (MF716697)	0.00	0.00	0.01	0.01	0.00	0.01	0.01	0.00							
*A. veronii* strain v4 (MF629678)	0.00	0.00	0.00	0.00	0.00	0.00	0.00	0.00	0.00						
*A. veronii* strain BLB‐01 (MF370515)	0.00	0.00	0.01	0.01	0.01	0.01	0.01	0.00	0.01	0.00					
*A. veronii* strain PYLGC (KY767542)	0.00	0.00	0.00	0.00	0.00	0.00	0.00	0.00	0.00	0.00	0.00				
*A. veronii* strain AnguAV2 (PP095387)	0.00	0.00	0.01	0.00	0.01	0.00	0.00	0.00	0.01	0.00	0.01	0.00			
*Bacillus subtilis* strain MRS3 (KP659617)	0.29	0.29	0.29	0.29	0.29	0.29	0.29	0.29	0.29	0.29	0.29	0.29	0.29		
*B. subtilis* strain MRS11 (KP659618)	0.28	0.28	0.28	0.28	0.29	0.28	0.28	0.28	0.29	0.28	0.28	0.28	0.28	0.00	

### 3.4 Histopathological Examination

**FIGURE 5:**
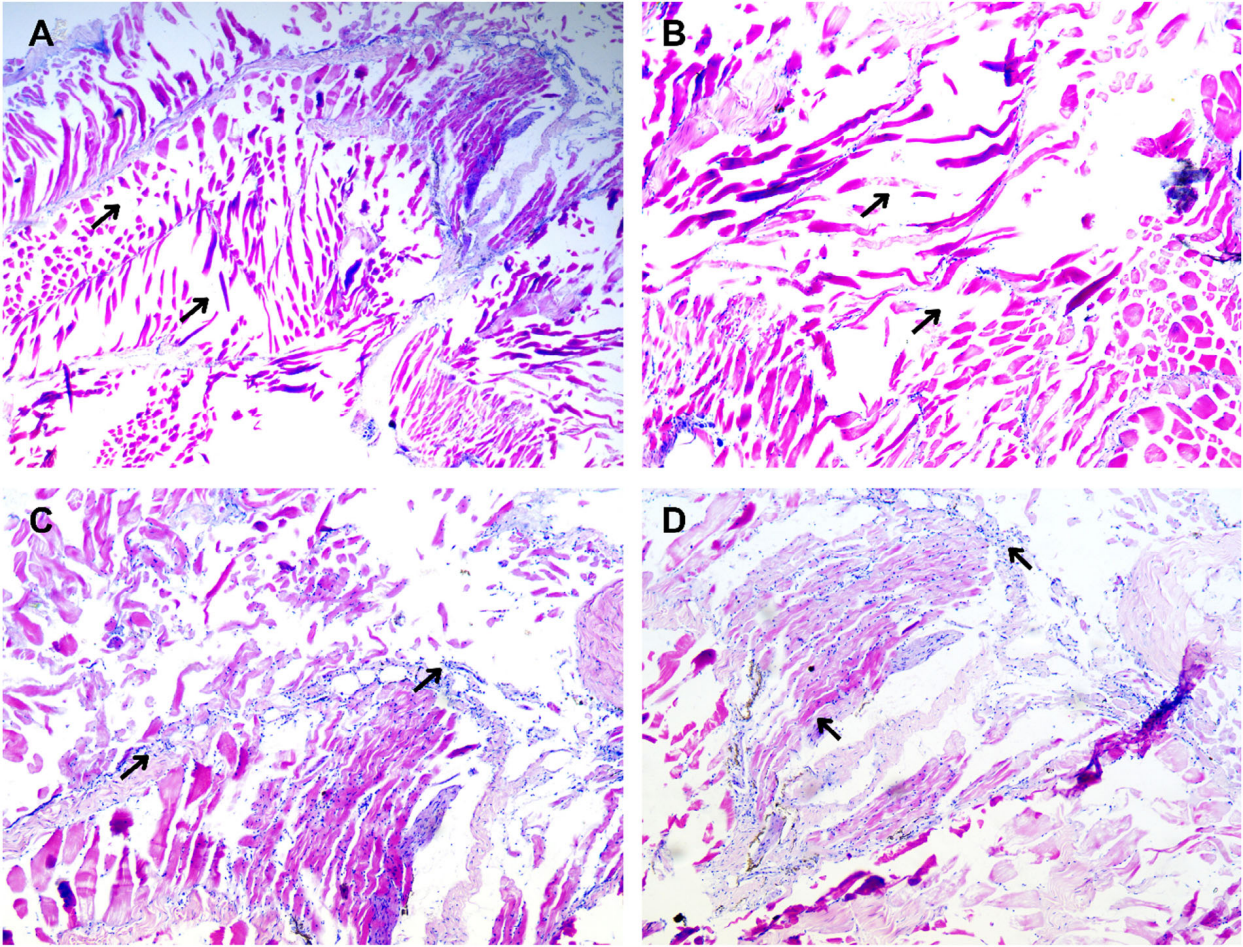
Histopathological alterations (muscle) in diseased Tilapia (*Oreochromis niloticus*). (A) Cellular degeneration and atrophy of muscle fibres (H&E, ×10). (B) Loss of cellularity, significant size variation (H&E, ×10). (C) Lymphocytic infiltration the arrow showing monocyte, intermuscular oedema (H&E, ×4). (D) Mononuclear inflammatory cells in muscle (black head arrow) (H&E, ×4).

The study investigated histopathological changes in infected *O. niloticus* muscle tissue. These histological changes are typically characterised by the presence of inflammatory cells, cellular degeneration and tissue necrosis (Figure 5A,B). Furthermore, muscle histology revealed a decrease in cellularity and evident lymphocytic infiltration, as indicated by the presence of monocytes (Figure 5C,D).

### 3.5 Antibiotic Susceptibility Analysis

VITEK 2 compact system AST‐GN cards were used to determine the susceptibility of *A. veronii* isolates to antimicrobial agents (Table 4). The study of antibiotic sensitivity for *A. veronii* revealed that the bacterium is highly sensitive to several antibiotics. The bacterium is most sensitive to the carbapenem group. Meropenem shows the highest sensitivity with an MIC of ≤ 0.25 µg/mL, and Imipenem also demonstrates strong sensitivity with an MIC of ≤ 0.5 µg/mL. Among the antibiotics tested, the β‐lactam/β‐lactamase inhibitor combinations and cephalosporins also show high sensitivity. On the other hand, the folate pathway inhibitor group shows the lowest sensitivity. *A. veronii* exhibits resistance to trimethoprim/sulphamethoxazole with an MIC of ≥ 320 µg/mL.

**TABLE 4:** In vitro antimicrobial susceptibility and minimum inhibitory concentration (MIC) patterns of *Aeromonas veronii* isolated from infected tilapia (*Oreochromis niloticus*).

Antibiotics	Sensitivity of *A. veronii* (n‐4)	MIC (µg/mL)	MIC range
Piperacillin/tazobactam	S	≤ 4	≤ 0.25–≥ 128
Cefuroxime	S	≤ 1	≤ 1–≥ 64
Ceftriaxone	S	≤ 0.25	≤ 1–≥ 64
Cefoperazone/Sulbactam	S	≤ 8	≤ 4–≥ 64
Cefepime	S	≤ 0.12	≤ 1–32
Imipenem	S	≤ 0.5	≤ 1–≥ 16
Meropenem	S	≤ 0.25	≤ 0.25–≥ 16
Amikacin	S	4	≤ 2
Gentamicin	S	≤ 1	≤ 1
Ciprofloxacin	S	1	≤ 0.25–≥ 4
Trimethoprim/sulphamethoxazole	R	≥ 320	≤ 20–≥ 320

Abbreviations: R, resistant; S, susceptible.

## 4 DISCUSSION

Bacterial diseases pose a significant threat to the global aquaculture industry. Bacterial infections in fish are opportunistic, causing illness when fish are stressed by poor water quality, overcrowding, or nutritional deficiencies (Noga 2010). Among these pathogens, *Aeromonas* spp. are frequently responsible for septicaemic infections in commercially cultured species (Beaz‐Hidalgo and Figueras 2013). In commercial tilapia culture, *A. veronii* has been identified as a highly virulent pathogen (Aly et al. 2023).

The study examined different clinical signs in infected fish, including bilateral exophthalmia, distended abdomens and haemorrhagic septicaemia. These observations are consistent with several findings. Raj et al. (2019), reported mass mortality and bilateral exophthalmia in cultured *O. niloticus* in India. Similarly, Bakry and Emeish (2022), found that *A. veronii* caused haemorrhagic septicaemia in cultured Nile tilapia in Egypt. Das et al. (2023) documented *A. veronii* infections in *Channa punctata* from Assam, India, where the fish exhibited bacterial septicaemic syndrome with ulcerations and mortality. In addition, European seabass (*Dicentrarchus labrax*) (Aboyadak et al. 2024) and ornamental fish species in China (Song et al. 2017) infected with *A. veronii* showed similar signs such as external haemorrhages, exophthalmia, cataracts, scale desquamation, skin ulcers and fin erosion. Furthermore, the biochemical findings of the studied isolates were also confirmed in accordance with earlier studies (Wassif 2018; Mzula et al. 2019).

The 16S rRNA genes contain both conserved and variable regions. The conserved regions reflect the phylogenetic relationships among species and act as sites for PCR priming (Smith et al. 2008). By analysing the 16S rRNA region through multiple sequence alignment and phylogenetic analysis, the isolated species was confirmed as *A. veronii*. The results of the phylogenetic analyses clearly indicate that similar samples of *A. veronii* are grouped into one genetic lineage, which is supported by high bootstrap values. Phylogenetic analysis is increasingly important in all fields of bacterial taxonomy (Acinas et al. 2004) as it traces lineages and classifies organisms within a broader context of their evolutionary connections (Pedersen et al. 2006). The low genetic distance highlights the potential geographic distribution and genetic conservation of the strain, which could have important implications for understanding its pathogenicity and spread in aquaculture systems globally.

The antibiogram profiling of *A. veronii* isolates revealed complete resistance to trimethoprim/sulphamethoxazole, while they were highly susceptible to β‐lactam/β‐lactamase inhibitor combinations and cephalosporins. In different studies, it is observed that *A. veronii* found in Tilapia exhibited resistance to oxytetracycline, enrofloxacin and amoxicillin (dos Santos et al. 2023); trimethoprim–sulphamethoxazole and erythromycin (Pauzi et al. 2020); and ampicillin and amoxicillin (Hassan et al. 2017; Aly et al. 2023). In addition, Nawaz et al. (2006), observed tetracyclineresistant aeromonads in farmraised catfish. Moreover, Elgendy et al. (2024) found that *Aeromonas* isolated from infected tilapia showed high resistance to amoxicillin, ampicillin and gentamicin while being susceptible to ciprofloxacin and florfenicol.

The study observed Tilapia infected with the *A. veronii* experience muscle changes, such as variations in size, degeneration, atrophy of muscle fibres, proliferation of mononuclear inflammatory cells between muscle bundles, and lymphocytic infiltration. These changes lead to tissue damage and impaired muscle function. These pathological findings are consistent with previous studies, which reported hypertrophy, swollen myofibres (Shahid et al. 2022) and epidermal necrosis accompanied by an influx of inflammatory cells into the dermis and hypodermis (Aly et al. 2023).

It has been observed that disease outbreaks are more common in fish raised under intensive culture conditions. Several factors exacerbate the infections and mortality rates, including high‐density production (Debnath et al. 2023), waste pollution, rough handling, temperature fluctuations and biofouling (Oyebola and Fada 2020). Fluctuations in salinity, pH, dissolved oxygen and water conductivity can also increase aeromonad infection in fish (Mzula et al. 2019). During the mortality occurance at our studied Shitalakshya River commercial cage culture farm, we identified poor water quality parameters, including a sharp decrease in dissolved oxygen and elevated industrial pollutants. Multiple studies have documented the risk to aquatic life in the Shitalakshya River (Islam et al. 2015). Many dyeing and textile industries in this area lack effluent treatment plants and discharge untreated wastedirectly into the Shitalakshya River, causing water pollution (Sharmin et al. 2020).

Overall, bacterial pathogen *A. veronii*, may causes a significant threat to tilapia aquaculture through mass mortality under poor water quality and intensive farming conditions. So enhancing water quality, implementing biosecurity measures and alternative treatments could significantly reduce disease outbreaks, and improve the sustainability and productivity of the tilapia aquaculture farms (Elgendy et al. 2024).

## 5 CONCLUSION

This study aimed to investigate the bacterial isolates present in infected tilapia cultured in cage units along the Shitalakshya River in Bangladesh. Clinical and histopathological examinations revealed abdominal distension, eye distension, muscle necrosis, hypertrophy and inflammation. The bacterial species *A. veronii* was identified based on colony morphology, biochemical characteristics and 16S rRNA sequencing. Moreover, detailed studies are needed to understand the potential reasons for the mortality of tilapia in open‐water commercial cage culture systems.

## Author Contributions

Conceptualization, methodology, software and formal analysis: **Sayed Mashequl Bari**. Data curation: **Mohammad Muttakinul Islam** and **Aktia Amina**. Writing–original draft preparation: **Sayed Mashequl Bari**. Writing–review and editing: **Marufa Khatun** and **A. M. Shahabuddin**. All authors have read and agreed to the published version of the manuscript.

## Ethics Statement

This study was carried out in accordance with the guidelines of the Sher‐e‐Bangla Agricultural University animal ethics ommittee and in accordance with local laws and regulations.

## Conflicts of Interest

The authors declare no conflicts of interest.

### Peer Review

The peer review history for this article is available at https://www.webofscience.com/api/gateway/wos/peer-review/10.1002/vms3.70103.

## Supporting information



Supporting information

## Data Availability

All data generated for this study are included in the article and Supporting Information.
