# Supplementary material for: Molecular Identification, Histopathology and Antibiotic Susceptibility Profiling of Aeromonas veronii Isolated from Oreochromis niloticus in Bangladesh
Source: Vet Med Sci. 2024 Nov 5;10(6):e70103. doi: 10.1002/vms3.70103 (PMC11536350; doi:10.1002/vms3.70103)
Supplement: Supplementary file 1 — Supporting information [file VMS3-10-e70103-s001.docx]

**Table. Maximum Likelihood fits of 24 different nucleotide substitution models.**

Model Parameters BIC AICc lnL (+I) (+G) R f(A) f(T) f(C) f(G) r(AT) r(AC) r(AG) r(TA) r(TC) r(TG) r(CA) r(CT) r(CG) r(GA) r(GT) r(GC)

HKY 31 6895.553 6648.912 -3293.409 n/a n/a 0.87 0.246 0.197 0.231 0.326 0.053 0.062 0.149 0.067 0.106 0.088 0.067 0.090 0.088 0.113 0.053 0.062

TN93 32 6902.010 6647.415 -3291.658 n/a n/a 0.87 0.246 0.197 0.231 0.326 0.053 0.062 0.128 0.066 0.130 0.087 0.066 0.111 0.087 0.096 0.053 0.062

HKY+I 32 6905.324 6650.730 -3293.315 0.46 n/a 0.95 0.246 0.197 0.231 0.326 0.051 0.060 0.157 0.064 0.111 0.085 0.064 0.095 0.085 0.118 0.051 0.060

HKY+G 32 6906.418 6651.823 -3293.861 n/a 1.51 0.90 0.246 0.197 0.231 0.326 0.052 0.061 0.152 0.066 0.108 0.087 0.066 0.092 0.087 0.115 0.052 0.061

TN93+I 33 6912.559 6650.011 -3291.952 0.43 n/a 0.93 0.246 0.197 0.231 0.326 0.051 0.060 0.130 0.064 0.137 0.084 0.064 0.117 0.084 0.098 0.051 0.060

T92 29 6912.564 6681.830 -3311.874 n/a n/a 0.88 0.222 0.222 0.278 0.278 0.059 0.074 0.131 0.059 0.131 0.074 0.059 0.104 0.074 0.104 0.059 0.074

TN93+G 33 6913.008 6650.460 -3292.177 n/a 1.51 0.91 0.246 0.197 0.231 0.326 0.052 0.060 0.129 0.065 0.134 0.086 0.065 0.115 0.086 0.097 0.052 0.060

HKY+G+I 33 6913.932 6651.384 -3292.639 0.30 0.92 1.01 0.246 0.197 0.231 0.326 0.050 0.058 0.161 0.062 0.114 0.082 0.062 0.097 0.082 0.122 0.050 0.058

T92+I 30 6917.430 6678.742 -3309.327 0.43 n/a 0.94 0.222 0.222 0.278 0.278 0.057 0.071 0.136 0.057 0.136 0.071 0.057 0.108 0.071 0.108 0.057 0.071

T92+G 30 6918.249 6679.561 -3309.736 n/a 1.51 0.91 0.222 0.222 0.278 0.278 0.058 0.072 0.134 0.058 0.134 0.072 0.058 0.106 0.072 0.106 0.058 0.072

TN93+G+I 34 6921.248 6650.747 -3291.317 0.16 0.68 0.99 0.246 0.197 0.231 0.326 0.050 0.058 0.132 0.062 0.143 0.082 0.062 0.122 0.082 0.100 0.050 0.058

K2 28 6922.480 6699.699 -3321.811 n/a n/a 0.88 0.250 0.250 0.250 0.250 0.067 0.067 0.117 0.067 0.117 0.067 0.067 0.117 0.067 0.117 0.067 0.067

JC 27 6924.874 6710.047 -3327.988 n/a n/a 0.50 0.250 0.250 0.250 0.250 0.083 0.083 0.083 0.083 0.083 0.083 0.083 0.083 0.083 0.083 0.083 0.083

GTR 35 6925.932 6647.479 -3288.680 n/a n/a 0.88 0.246 0.197 0.231 0.326 0.072 0.037 0.128 0.090 0.130 0.081 0.039 0.111 0.097 0.097 0.049 0.069

T92+G+I 31 6926.039 6679.398 -3308.652 0.29 0.90 1.02 0.222 0.222 0.278 0.278 0.055 0.069 0.141 0.055 0.141 0.069 0.055 0.112 0.069 0.112 0.055 0.069

K2+I 29 6927.066 6696.331 -3319.124 0.47 n/a 0.96 0.250 0.250 0.250 0.250 0.064 0.064 0.123 0.064 0.123 0.064 0.064 0.123 0.064 0.123 0.064 0.064

K2+G 29 6928.090 6697.356 -3319.637 n/a 1.51 0.91 0.250 0.250 0.250 0.250 0.065 0.065 0.119 0.065 0.119 0.065 0.065 0.119 0.065 0.119 0.065 0.065

GTR+I 36 6931.473 6645.068 -3286.471 0.43 n/a 0.94 0.246 0.197 0.231 0.326 0.075 0.032 0.131 0.094 0.137 0.073 0.034 0.117 0.096 0.099 0.044 0.068

GTR+G 36 6931.796 6645.390 -3286.632 n/a 1.51 0.91 0.246 0.197 0.231 0.326 0.074 0.034 0.129 0.092 0.134 0.077 0.036 0.114 0.097 0.097 0.047 0.068

JC+I 28 6931.992 6709.211 -3326.567 0.54 n/a 0.50 0.250 0.250 0.250 0.250 0.083 0.083 0.083 0.083 0.083 0.083 0.083 0.083 0.083 0.083 0.083 0.083

JC+G 28 6934.789 6712.009 -3327.966 n/a 1.50 0.50 0.250 0.250 0.250 0.250 0.083 0.083 0.083 0.083 0.083 0.083 0.083 0.083 0.083 0.083 0.083 0.083

K2+G+I 30 6935.941 6697.253 -3318.582 0.30 0.92 1.01 0.250 0.250 0.250 0.250 0.062 0.062 0.126 0.062 0.126 0.062 0.062 0.126 0.062 0.126 0.062 0.062

JC+G+I 29 6940.479 6709.745 -3325.831 0.31 0.84 0.50 0.250 0.250 0.250 0.250 0.083 0.083 0.083 0.083 0.083 0.083 0.083 0.083 0.083 0.083 0.083 0.083

GTR+G+I 37 6942.966 6648.609 -3287.238 0.12 0.68 0.98 0.246 0.197 0.231 0.326 0.077 0.030 0.132 0.096 0.141 0.069 0.032 0.121 0.095 0.099 0.042 0.067
